# Intrauterine Growth Retardation and Nonalcoholic Fatty Liver Disease in Children

**DOI:** 10.1155/2011/269853

**Published:** 2011-11-30

**Authors:** Anna Alisi, Nadia Panera, Carlo Agostoni, Valerio Nobili

**Affiliations:** ^1^Liver Research Unit, Bambino Gesù Children's Hospital and Research Institute, 00165 Rome, Italy; ^2^Department of Maternal and Pediatric Sciences, Fondazione IRCCS Ca' Granda—Ospedale Maggiore Policlinico, University of Milan, 20122 Milan, Italy

## Abstract

Intrauterine growth retardation (IUGR), the most important cause of perinatal mortality and morbidity, is defined as a foetal growth less than normal for the population, often used as synonym of small for gestational age (SGA). Studies demonstrated the relationships between metabolic syndrome (MS) and birthweight. This study suggested that, in children, adolescents, and adults born SGA, insulin resistance could lead to other metabolic disorders: type 2 diabetes (DM2), dyslipidemia, and nonalcoholic fatty liver disease (NAFLD). NAFLD may evolve to nonalcoholic steatohepatitis (NASH), and it is related to the development of MS. Lifestyle intervention, physical activity, and weight reduction represent the mainstay of NAFLD therapy. In particular, a catch-up growth reduction could decrease the risk to develop MS and NAFLD. In this paper, we outline clinical and experimental evidences of the association between IUGR, metabolic syndrome, insulin resistance, and NAFLD and discuss on a possible management to avoid the risk of MS in adulthood.

## 1. Introduction

Intrauterine growth retardation (IUGR) is one of the most important causes of perinatal mortality and morbidity and affects approximately 7–15% of worldwide pregnancies [1]. IUGR is commonly defined as foetal growth less than that characterizing commonly healthy population. However, to date, there is no a clear internationally conventional clinical definition for this term. In fact, although some studies use IUGR as synonym of small for gestational age (SGA), it is important to remember that infants born with IUGR may or may not be necessarily SGA and, similarly, infants who are SGA may be born without growth-restricting processes characterizing IUGR [2, 3].

Although several causes or risk factors have been suggested for development of IUGR, including those of maternal, placental, and foetus origin [4], it is very difficult to establish, in most situations, the real cause of this condition.

In addition to the high rates of perinatal mortality, IUGR, recently, has been often associated with the development of several features of metabolic syndrome (MS) in adulthood, increasing seriously the risk of mortality associated to cardiovascular disease [5]. Features of MS classically associated with IUGR are insulin resistance, hypertension, dyslipidemia, impaired glucose tolerance, and type 2 diabetes (DM2); but, very recently, also nonalcoholic fatty liver disease (NAFLD) has been included among the persistent IUGR-dependent metabolic dysfunctions [6, 7].

Furthermore, on the basis of numerous pieces of evidences demonstrating that early improvement in growth appears beneficial for a number of important outcomes, the personnel of neonatal follow-up clinics are encouraged to promote early catch-up growth for SGA subjects [8]. However, from 2003, several controversial studies have forwarded the hypothesis that restricting postnatal catchup after prematurity could prevent later metabolic abnormalities [9].

In this paper, we overview on several aspects associated to IUGR, discussing with a particular attention all correlation with MS and NAFLD. In addition, here, we emphasize on the fact that an optimal nutritional management needs to achieve normal growth variables and a normal body composition without increasing the risk of MS in adulthood.

## 2. Concepts of IUGR and SGA

IUGR and SGA are related but not synonymous, although both the terms are used interchangeably and both denote malnutrition. 

IUGR is a clinical condition that occurs when the unborn baby is at or below the 10th weight percentile for his or her age (in weeks). The foetus is affected by a pathologic restriction in its ability to grow due to anatomical and/or functional disorders and diseases in the fetoplacental-maternal system [10]. A satisfactory definition of IUGR has been suggested by the American College of Obstetricians and Gynecologists (ACOG) as “a foetus that fails to reach his potential growth” [11]. IUGR is usually classified into symmetric when growth restriction implies that head circumference, length, and weight are proportionally SGA; asymmetrical when head circumference is appropriate for gestational age (AGA), whereas length and weight are reduced. Symmetric IUGR may depend on a problem during early development and it is associated with causes that affect total foetal cell number, including chromosomal, genetic, teratogenic, intra-uterine infections and severe hypertensive aetiologies. Asymmetrical IUGR is often of a later onset, and occurs whether an undernourished foetus directs most of its energy to maintain growth of vital organs, such as the brain and heart, at the expense of the liver, muscle, and fat. This type of IUGR is associated with poor maternal nutrition or late onset exacerbation of maternal vascular disease (preeclampsia, chronic hypertension) and represents an adaptation to an unfavourable intrauterine environment [12].

SGA indicates that a foetus or neonate is below a reference range for size or weight for a given gestational age. It is a statistical definition used for newborns whose birthweight is less than 10th percentile for that particular gestational age, referring to the weight of the infant at birth and not to the growth pattern. Thus, SGA may reflect a normal pattern in a given population, but for definition often stackable to IUGR [12, 13].

## 3. Epidemiology of IUGR

IUGR is an important clinical problem being the most important cause of perinatal morbidity and mortality second only to prematurity. IUGR affects approximately 7–15% of pregnancies [1]. The prevalence is estimated to be approximately 8% in the general population. It has been reported that 52% of unexplained stillbirths are associated with IUGR, which is also the cause of 10% perinatal mortality. Furthermore, up to 72% of unexplained foetal deaths are associated with SGA below the 10th percentile. Based on available data, it has been estimated that between 2.3 and 10% of all infants are born SGA, although this may still be a gross underestimate in global terms [3].

## 4. Risk Factors of IUGR

During the last ten years, several studies, understanding of IUGR-associated pathophysiology, have been launched. Actually we know many causes responsible of IUGR, that are usually summarized in three types of risk factors: maternal, foetal, and placental factors (Table 1).

The mother's nutritional status is the major determinant of IUGR. This includes maternal malnutrition before conception or insufficient nutritional intake during pregnancy. Furthermore, smoke, substance abuse like alcohol and drug, maternal hypertension, frequent pregnancies, multiple pregnancy, anaemia, and chronic maternal diseases of heart, kidneys, lungs, or liver, may have an adverse effect on foetal weights [4]. Chronic maternal disease alters normal regulation of hormonal activity during pregnancy resulting in increased levels of free circulating corticotropin-releasing hormone (CRH) before its normal increase at term [14].

The foetal abnormalities, such as chromosomal defects, congenital malformations, chromosomal aberrations, and infections, can be crucial consequences of IUGR. Intrauterine foetal infections, for example, can limit foetal growth by directly damaging the foetal brain and neuroendocrine axis that support foetal growth via insulin-like growth factors (IGFs) and insulin and by damaging the foetal heart, leading to diminished cardiac output, poor placental perfusion, and inadequate nutrient substrate uptake [15].

The placental transport is the major player in foetal nutrition as it determines the availability of oxygen and nutrients to the foetus. The placenta should be considered as a sensor between maternal nutritional, metabolic, endocrine and vascular conditions and foetal requirements. Placental insufficiency in IUGR is associated with a limited maternal-foetal nutrient exchange as the normal supply of growth promoting hormones to the foetus (placental lactogen, steroid hormones, insulin-like growth factors) [15, 16]. 

Vascular placental pathology which is a significant leading factor in IUGR, is often associated with aetiologies of chronic maternal diseases like hypertension, autoimmune diseases, obesity and diabetes [17]. 

The role of oxidative stress and proinflammatory cytokines is still under exploration; however, it is suggested that the oxidative stress could cause vascular dysfunction in the placenta leading to foetal compromise [18].

## 5. Consequences of IUGR

Infants exposed to IUGR are not only at risk for an increase in many perinatal morbidities, but they have been also associated with adult disease in both human epidemiological studies and in animal models [19, 20].

The acute neonatal consequences of IUGR are perinatal asphyxia, hypothermia, hypoglycaemia polycythemia and other neonatal adaptive problems. The sequelae of perinatal asphyxia include multiorgan dysfunction neonatal encephalopathy and metabolic acidemia [21].

Numerous epidemiological studies by Barker between 1980s and 1990s have demonstrated a strong association between a low birth weight, adult obesity, diabetes, and cardiovascular disease [22]. 

Recently, many other studies have confirmed the correlation between IUGR newborns and the development of the MS later in life, comprising arterial hypertension, hypercholesterolemia, cardiovascular disease, impaired glucose tolerance, and/or DM2, and many other diseases, including osteoporosis. This association, described in various populations, is unrelated to age, sex, and ethnicity and occurs independently of weight and physical activity [5, 23].

**Figure 1:**
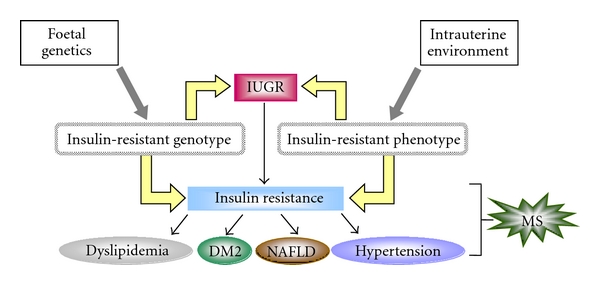
Possible hypotheses to explain the association between IUGR and MS. IUGR: intrauterine growth retardation; DM2: type 2 diabetes; NAFLD: nonalcoholic fatty liver disease; MS: metabolic syndrome.

From Barker et al. [2], who was the first to introduce the existing correlation between birth size and later development of MS in adult life, several authors [24] showed a wide collection of data highlighting that subjects born SGA are prone to central redistribution of adipose tissue and are at high risk for developing insulin resistance, DM2, MS, and cardiovascular disease. Although the mechanism able to induce MS in IUGR is still unclear in all observed cases, increased insulin resistance appeared to play a key role. Two theories have been proposed to explain the development of insulin resistance in IUGR: the first is the foetal reprogramming due to thrifty phenotype hypothesis; the second is the establishment of an insulin-resistant genotype independently of intrauterine environment (Figure 1).

 According to “thrifty phenotype hypothesis,” maternal undernutrition during pregnancy modifies the programming of biochemical mechanisms related to endocrine-metabolic control inducing permanent changes in glucose-insulin metabolism. These changes include reduced capacity for insulin secretion and insulin resistance which, combined with the effects of obesity, aging, and physical inactivity, may result in cardiovascular and metabolic complications [25]. Furthermore, it has been suggested that, in condition of undernutrition, a genotype conferring insulin resistance would be preferentially selected during evolution because this genotype would increase survival among small babies. This phenomenon is called the “surviving small baby hypothesis” [26]. This foetal programming of adaptation to an adverse intrauterine environment results in increased sensitivity of the peripheral tissues to metabolic hormones, such as glucocorticoids and insulin, this latter condition enhances survival and maximizes growth and fuel deposition, as the nutritional pattern improves after birth. So a “thrifty phenotype” postulates that the intrauterine deprivation programs the fetus to increase appetite and obesity, hypertension, and diabetes [19]. If postnatal nutrient availability is greater than prenatally predicted, enhanced postnatal growth and fat deposition will occur. In turn, this increased adiposity will lead to adult insulin resistance. Certainly, the risk of developing adult MS is the greatest when poor prenatal growth is coupled with rapid catch-up growth during childhood [27].

The alternative hypothesis to development of insulin-resistant phenotype in IUGR individuals has suggested that insulin resistance might be genetically determined independently of unfavourable intrauterine environment; as consequences, thus, also a genetic predisposition to metabolic consequences of IUGR. In particular, Hattersley and Tooke [3] proposed a “foetal insulin hypothesis,” suggesting the strong contribution of genetic factors to alter either foetal insulin secretion or sensitivity of foetal tissues. Polymorphisms or mutations in genes associated to insulin resistance could result in impaired foetal growth, low birthweight, and subsequent susceptibility to DM2 and cardiovascular disease in adult life. In fact, monogenic disorders affect foetal insulin secretion and resistance causing retarded foetal growth *in utero* during the third trimester, just when the insulin increase should act as one of the major growth factors in foetal life. Some monogenic disorders and their effects on insulin and birth weight are reported in Table 2. For example, mutations in the gene encoding glycolytic enzyme glucokinase have been observed, this mutation results in beta-cell dysfunction, low-birth-weight and DM2 susceptibility in childhood and adulthood [28]. However, the monogenic diseases are rare and thus they cannot explain the low birth weight case normally seen. So it should be clear that both genetic and environmental factors and their possible interactions may contribute to the development of the MS in later life [29].

## 6. Evidence of Association between SGA and MS

There are several articles that demonstrated the association between SGA and features of MS. 

Interestingly, there are three relevant clinical trials demonstrating a strong association between low birth weight and insulin resistance. In the first study, including 85 SGA subjects and 23 AGA subjects, the authors found a close link between insulin secretion/sensitivity, patterns of rapidity, and length of catch-up-growth process during early postnatal life [30]. In the second study, the authors found that mildly impaired insulin sensitivity in 79 prepubertal short children born SGA is associated with growth hormone treatment [31]. Finally, the most recent longitudinal study, conducted on 51 individuals, demonstrated that visceral fat excess was, in postcatch-up SGA children, already present at the age of 6 years and this increment was closely related to the increment in fasting insulin [32].

The programming is today the most appropriate term to describe the plasticity of the developing organs, eventually resulting in permanent changes in structure and/or function. Therefore, the major programmed defect of metabolism linking the adverse intrauterine environment is insulin resistance that, in all recent studies, is considered the key factor for the simultaneous development of the MS and the later occurrence of DM2 [33]. 

However, although adults born SGA have a higher incidence of metabolic risk factors (2.3%) than those born AGA (0.4%), there is no evidence that dyslipidemia occurs more commonly among children born SGA than in the normal childhood population [34]. Dyslipidemia, as well as DM2, might result from the initial development of insulin resistance. One interesting study showed that SGA children with poor catch-up growth in height may be at the highest risk for hypercholesterolemia [35]. The real mechanisms are still unclear, but some evidence suggests that association between insulin resistance and dyslipidemia in SGA subjects could be the consequence of genetic/environmental interactions acting during catch-up growth phase [24].

## 7. Definition of NAFLD

The term of NAFLD includes a spectrum of diseases ranging from simple fatty liver to nonalcoholic steatohepatitis (NASH) with or without fibrosis that may eventually progress to cirrhosis. Simple fatty liver remains a benign process in most affected people, while the presence of liver inflammation, typically observed in NASH, may be the driving force for the development cirrhosis and hepatocellular carcinoma. Histological assessment plays an important role in the diagnosis and management of paediatric NAFLD; thus, it is important a carefully discrimination among the different histological features of NAFLD. 

The main histological findings in paediatric NAFLD are simple steatosis, ballooning, inflammation and fibrosis, but also other liver lesions may be present. Despite the increased number of studies directed to search less invasive diagnostics methods, liver biopsy evaluation continues to be considered the “gold standard” in establishing the diagnosis as well the severity of NAFLD. However, liver biopsy presents several limitations including high risk, high costs, “sampling errors” and the interobserver variations in the histopathologic assessment [36–38].

## 8. Epidemiology of NAFLD

NAFLD is an increasingly recognized cause of liver disease worldwide. The incidence and the true prevalence of paediatric NAFLD remains unknown due to the lack of prospective studies. Population-based studies might provide more accurate figures, but few such studies have been reported to date. A high prevalence rate between 2% and 80% of NAFLD has been reported in North and South America, Europe, Australia, and Asia [38]. The high variability in prevalence data from different geographical areas not only may depend on the type of test but it may also be influenced by the age, sex and ethnicity-collected population. The importance of metabolic factors has been also demonstrated by several evidences suggesting that NASH might be considered the hepatic manifestation of the MS. In particular, DM2 is associated both with obesity, NAFLD, and development of progressive liver fibrosis. Dyslipidemia (i.e., hypertriglyceridemia and/or hypercholesterolemia), which is frequently associated with both obesity and DM2, has been also reported in 20–80% of patients with NASH. Interestingly, also hyperglycemia is associated with NASH; in fact, the prevalence of NAFLD rises in hyperglycemic patients, and insulin resistance is more severe in individuals with NASH in versus steatosis [38, 39].

## 9. Etiopathogenesis of NAFLD

**Figure 2:**
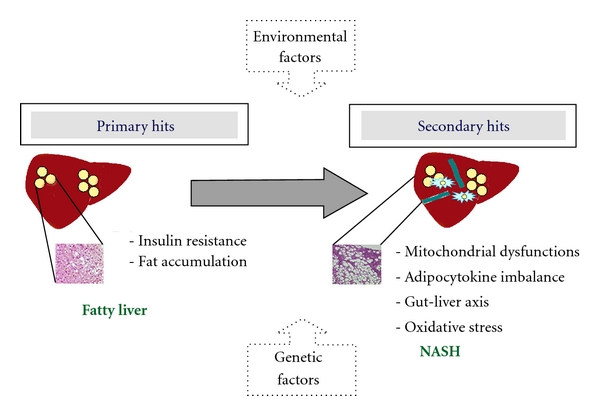
Simplified representation of NASH pathogenesis. NASH, nonalcoholic steatohepatitis.

The etiopathogenesis of NAFLD has been long disputed. Most authors consider plausible the theory of “multiple hits.” According to this theory, liver fat accumulation and insulin resistance, which are the suggested “primary hits,” may lead to simple steatosis and making fatty liver more vulnerable to possible “secondary hits” which may be involved in the progression to NASH (Figure 2). “Secondary hits” include oxidative stress, mitochondrial dysfunction, and imbalance of production/release of hormones derived from adipose tissue (adipocytokines) [40, 41]. More recently, also a gut/liver axis hypothesis has been included. This suggests that bacterial endotoxins of intestinal origin and the related mechanisms of innate immune response may act as possible inductors of necroinflammatory lesions in the progression of steatosis to NASH and severe fibrosis [42]. This is at present the most accredited pathogenetic model (Figure 2), even though, the complicated molecular network of interactions that leads to NAFLD/NASH often confuses causes and effects. Despite this apparent confusion, all today theories consider the insulin resistance the major actor in the development of paediatric NAFLD [43]. Although causative mechanism which links insulin resistance and NAFLD is still under investigation, two main hypotheses are formulated. In genetically predisposed individuals, environmental and nutritional factors interact with thrifty genes favouring the occurrence of whole body insulin resistance, and, in turn, the inappropriate accumulation of fats in muscle and liver, or causing fat deposition and consequent insulin resistance first in the liver and just in sequence at the peripheral sites [44].

## 10. Association between SGA an NAFLD and Possible Therapeutic Management

A number of recent studies shed light on the relationship between IUGR, rapid weight gain after birth, and increased risk of MS in adulthood [45–47]. These studies highlight the fact that the nutrient deficiency during fetal life can induce, consequently to an adaptive mechanism, an upregulation of the expression of insulin receptor. So after birth, the relevant activation of insulin signalling pathway could lead to a rapid weight gain [45].

Interestingly, Rueda-Clausen and colleagues observed that rats with hypoxia-induced IUGR and fed with high-fat diet were more susceptible to develop metabolic derangement of lipid homeostasis. This *in vivo model *of IUGR also induced insulin resistance, impaired glucose tolerance accompanied by augmented protein kinase C phosphorylation, both in the liver and in the skeletal muscle [46].

In another recent study, Magee and coworkers showed how IUGR might be associated with not only obesity and lipid abnormalities, but also fatty liver and inflammation. In this study, IUGR offsprings displayed hepatic downregulation of peroxisome proliferator-activated receptor (PPAR)*α* and *γ* and upregulation of sterol regulatory element-binding protein-1 and fatty acid synthase [47]. All these proteins are involved in the regulation of lipid metabolism and lipid-associated inflammatory response strongly and are often associated to NAFLD pathogenesis [40].

Finally, only one report describes the association between low birthweight and NAFLD in humans [7]. In this study, Nobili et al. demonstrated the association of paediatric NAFLD with IUGR, independently of insulin resistance. In 90 Italian children with biopsy-proven NAFLD, the prevalence of SGA with NAFLD was approximately fourfold higher if compared to the average SGA prevalence of children admitted to our hospital. As already described, insulin resistance may represent the link between metabolic/NAFLD and IUGR. Consequently, IUGR and especially low birth weight might represent an important risk factor for paediatric NAFLD.

Therapeutic approaches in NAFLD try to limit the progression from fatty liver to steatohepatitis and to reverse histological features of necroinflammation and fibrosis. Changes in lifestyle represent valuable means in both prevention and treatment of fatty liver and NASH. Weight loss and weight control can be the first line to prevent the onset of fatty liver, being overweight/obesity major contributing factors to fatty infiltration of the liver. In fact, diet and physical exercise are currently considered the cornerstone for paediatric NAFLD [38].

Singhal et al. [9] suggests that restricting postnatal catchup after prematurity will avoid later metabolic abnormalities. In addition, it has been suggested that the consumption of human milk has many benefits and an adequate breastfeeding protects against the development of obesity and NAFLD in childhood and adults [48]. Thus, even though it remains to be determined what are the mechanisms and the specific differences in composition between human milk and commercial formula able to produce metabolic disturbance, and how breastfeeding may exert its preventive action, it is clear that certainly caution should be used in nutrition program for IUGR individuals. In particular, catch-up growth should be restricted and also an accurate research of the “the best milk formula” should be made to prevent some features of MS.

## 11. Conclusions

In this paper, we overviewed principal characteristics of IUGR and the relationship between birth weight and some main features of MS, especially NAFLD.

In addition, we demonstrated that several evidence exists on the associations between low birth weight and some features of metabolic syndrome in later life. It appears that the period of maximal foetal growth and the later period of catch-up growth specifically influence insulin resistance and development of MS and its comorbidities. However, there are still significant gaps in the knowledge of mechanisms generating metabolic profile and outcome in IUGR individuals. 

As catch-up growth and nutrition are involved in development of metabolic abnormalities in SGA subjects, it may be possible to alter early metabolic programming by improving specific macro- or micronutrient deficiencies during the neonatal period. 

In conclusion, a routine health surveillance of all adults born SGA should be recommended in normal clinical practice, and an adequate lifestyle program (appropriate diet and exercise) might help to prevent MS and NAFLD also in these subjects with high risk for both diseases.

##  Conflict of Interests

The authors declared that they have no conflict of interests.

## Figures and Tables

**Table 1:** Risk factors in IUGR.

Origin	Risk factors
*Maternal causes*	

Medical conditions	

(i) Vascular diseases	Chronic hypertension
	Preeclampsia early in gestation
	Diabetes mellitus
	Systemic lupus erythematosus
	Chronic kidney disease
	Inflammatory bowel disease
	Severe lung disease
(ii) Infections	Syphilis
	Toxoplasmosis
	Cytomegalovirus
	Rubella virus
	Hepatitis B virus
	Herpes simplex virus 1 or 2
	HIV-1
	Helicobacter pylori
	Malaria

Social conditions	

(i) Malnutrition	Low prepregnancy weight and small maternal size
	Poor weight gain during pregnancy, especially in latter half
	Nutritional deficiencies: protein folic acid, vitamin A, B, C, zinc, calcium
(ii) Drugs use	Cigarettes, alcohol, heroin, cocaine
	Teratogens, antimetabolites, and therapeutic agents such as trimethadione, warfarin, and phenytoin
(iii) History	Recent pregnancy and/or high parity
	Multiple pregnancy
	Prior history of IUGR pregnancy
	Residing at altitude over 5,000 ft (1,500 m)

*Fetal Causes*	

Genetic factors	Race, ethnicity, nationality, sex parity (primiparous, weigh less than subsequent siblings), genetic disorders (Achondroplasia, Russell-Silver syndrome)
Chromosomal anomalies	Chromosomal deletions
	Trisomy 13,18, and 21
Congenital malformations	Anencephaly, GI atresia, Potter's syndrome, and pancreatic agenesis

*Placental Causes*	

Placental insufficiency	Reduced blood flow
Anatomic problems	Multiple infarcts
	Aberrant cord insertions
	Umbilical vascular thrombosis and hemangiomas
	Premature placental separation
	Small Placenta

**Table 2:** Monogenic defects associated with insulin-resistant dependent IUGR.

Genetic	Defects	Disease	Pathophysiology
Glucokinase (GK)	Heterozigygous mutations	Glucokinase defiency	Low foetal insulin secretion
Insulin promoter factor 1 (IPF1)	Homozigygous mutation	Pancreatic agenesis	Foetal insulin secretion is abolished
Suplphonylurea-receptor1/Kir6 (SUR1/Kir6)	Homozigygous mutation	Nesidioblastosis	Increased insulin secretion
Insulin receptor (IR)	Homozigygous mutation	Leprechaum syndrome	Marked insulin resistance
6q22-q33	Duplication or Paternal isodisomy	Transient neonatal diabetes	Reduced insulin secretion
